# CSF sphingolipids are correlated with neuroinflammatory cytokines and differentiate neuromyelitis optica spectrum disorder from multiple sclerosis

**DOI:** 10.1136/jnnp-2024-333774

**Published:** 2024-06-06

**Authors:** Lisa Shi, Laura Ghezzi, Chiara Fenoglio, Anna Margherita Pietroboni, Daniela Galimberti, Francesca Pace, Todd A Hardy, Laura Piccio, Anthony S Don

**Affiliations:** 1 School of Medical Sciences, Charles Perkins Centre, and Brain and Mind Centre, The University of Sydney, Sydney, New South Wales, Australia; 2 Department of Biomedical, Surgical and Dental Sciences, University of Milan, Milan, Italy; 3 La Fondazione IRCCS Ospedale Maggiore Policlinico, Milano, Italy; 4 Department of Neurology, Washington University in St Louis, St Louis, Missouri, USA; 5 Department of Clinical-Surgical Diagnostic and Pediatric Sciences, University of Pavia, Pavia, Lombardia, Italy; 6 Concord Hospital, Department of Neurology, The University of Sydney, Sydney, New South Wales, Australia

**Keywords:** MULTIPLE SCLEROSIS, BIOCHEMISTRY, CSF

## Abstract

**Background:**

There is a need for biomarkers of disease progression and therapeutic response in multiple sclerosis (MS). This study aimed to identify cerebrospinal fluid (CSF) lipids that differentiate MS from other neuroinflammatory conditions and correlate with Expanded Disability Status Scale (EDSS) scores, gadolinium-enhancing lesions or inflammatory mediators.

**Methods:**

Lipids and inflammatory cytokines/chemokines were quantified with liquid chromatography-tandem mass spectrometry and multiplex ELISA, respectively, in CSF from people with untreated MS, neuromyelitis optica spectrum disorder (NMOSD), other inflammatory neurological diseases and non-inflammatory neurological diseases (NIND). Analytes were compared between groups using analysis of variance, and correlations were assessed with Pearson’s analysis.

**Results:**

Twenty-five sphingolipids and four lysophosphatidylcholines were significantly higher in NMOSD compared with MS and NIND cases, whereas no lipids differed significantly between MS and NIND. A combination of three sphingolipids differentiated NMOSD from MS with the area under the curve of 0.92 in random forest models. Ninety-four lipids, including those that differentiated NMOSD from MS, were positively correlated with macrophage migration inhibitory factor (MIF) and 37 lipids were positively correlated with CSF protein in two independent MS cohorts. EDSS was inversely correlated with cholesterol ester CE(16:0) in both MS cohorts. In contrast, MIF and soluble triggering receptor expressed on myeloid cells 2 were positively associated with EDSS.

**Conclusions:**

CSF sphingolipids are positively correlated with markers of neuroinflammation and differentiate NMOSD from MS. The inverse correlation between EDSS and CE(16:0) levels may reflect poor clearance of cholesterol released during myelin break-down and warrants further investigation as a biomarker of therapeutic response.

**WHAT IS ALREADY KNOWN ON THIS TOPIC**

- Although prior analyses of the cerebrospinal fluid (CSF) lipidome have identified changes to specific lipid species in the CSF of people with multiple sclerosis (MS), there are currently no validated lipid biomarkers that differentiate MS from related neuroinflammatory diseases, or correlate with clinical disease severity scores.

**WHAT THIS STUDY ADDS**

- First, we established that a broad range of sphingolipids are substantially higher in the CSF of people with neuromyelitis optica spectrum disorder (NMOSD) compared with MS and non-inflammatory neurological diseases, and that these sphingolipids are strongly associated with the cytokine macrophage migration inhibitory factor. Second, we demonstrate that CSF cholesterol ester (16:0) was reproducibly and inversely correlated with Expanded Disability Status Scale scores in two independent MS cohorts.

**HOW THIS STUDY MIGHT AFFECT RESEARCH, PRACTICE OR POLICY**

- Pending validation in future studies, high levels of particular CSF sphingolipids could aid in differentiating NMOSD from MS during diagnosis, and these sphingolipids may be useful for tracking disease activity and therapeutic response in NMOSD cases. On the other hand, CSF cholesterol ester (16:0) could be used as a biomarker for therapeutic efficacy in clinical trials of people with MS.

## Introduction

 Multiple sclerosis (MS) diagnosis relies on a combination of clinical presentation and the presence of white matter lesions disseminated in time and space on MRI.^1^ Oligoclonal immunoglobulin G (IgG) bands (OCBs) in the cerebrospinal fluid (CSF) provide additional diagnostic value but are not present in all cases and are not unique to MS. In some cases, MS can be difficult to differentiate from other neurological disorders with overlapping clinical presentation.^2^ For example, optic neuritis, myelitis and grey and white matter lesions are characteristic of both MS and neuromyelitis optica spectrum disorder (NMOSD).^3 4^ Serum antibodies to the astrocyte water channel aquaporin-4 (AQP4) are specific to NMOSD and permit a positive diagnosis.^4^ However, these antibodies are absent in 20–40% of NMOSD cases,^5^ leaving a gap in diagnostic sensitivity. Importantly, treatment of NMOSD with some MS therapeutics can exacerbate the disease.^3^

Relapses and disease progression are diagnosed through assessment of new symptoms and MRI lesions, however, lesion load correlates weakly with clinical severity.^6^ The discovery of fluid biomarkers that provide early detection of disease activity and prediction of therapeutic response would enable more timely intervention and guide therapeutic choice.^7^ Candidate biomarkers include neurofilament light chain (NfL), an axonal protein that is released by degenerating neurons; chitinase-3-like-1 (CHI3L1), expressed by activated astrocytes, microglia and macrophages^8^; and the C-X-C motif chemokine ligand 13 (CXCL13), which regulates B-cell migration and organisation within lymphoid organs.^9 10^ Higher serum NfL levels at baseline predict faster disability progression, brain volume loss and accrual of new T2 lesions on MRI, adding to the specificity and sensitivity of current models for predicting disease progression.^11 12^ However, its levels increase with age and decrease with body mass index (BMI), making it difficult to set cut-off values.^12^ Similarly, CHI3L1 levels are higher in the CSF, serum and plasma of people with MS and are associated with Expanded Disability Status Scale (EDSS), relapse rate, MRI lesion load and prognosis.^8 13^ These biomarkers reflect neurodegenerative or inflammatory processes and are not specific to MS or demyelinating diseases.

Myelin is composed of 70–80% (dry weight) of lipids, and within the central nervous system (CNS), the lipids galactosylceramide (GalCer) and sulfatide (ST) are synthesised exclusively by oligodendrocytes, highly abundant in myelin and essential for myelin stability.^14^,^17^ We hypothesised that the CSF lipidome will differ substantially in demyelinating diseases such as MS and NMOSD, compared with other neuroinflammatory diseases. Prior studies using thin-layer chromatography have demonstrated increased ST but not GalCer in the CSF of people with MS,^18^ whereas more recent studies using liquid chromatography-tandem mass spectrometry have shown increased ceramide (Cer) and hexosylceramide (HexCer) (in the CNS, HexCer is almost entirely GalCer),^16^ but no change to ST.^19^,^22^ Others have reported increased C18 and C20 fatty acids, and decreased lysophosphatidylcholine (LPC) and sphingomyelin (SM) in MS relative to other neurological diseases.^23 24^ In one study C16 HexCer was positively correlated with EDSS scores,^22^ while C16 lysophosphatidylinositol was negatively correlated in another.^25^ No prior studies have compared the CSF lipidome between MS and NMOSD.

In this study we aimed to identify CSF lipids that (1) correlate with EDSS scores, gadolinium-enhancing (Gd+) lesions and inflammatory cytokines in people with MS; and (2) distinguish MS from NMOSD, other inflammatory neurological diseases (OIND) and non-inflammatory neurological diseases (NIND).

## Materials and methods

### CSF samples

Discovery cohort samples were from the Washington University Multiple Sclerosis and Nervous System Diseases Repository and Database. Validation cohort samples were obtained from the IRCCS Ospedale Maggiore Policlinico, Milan. Informed consent was obtained from all participants prior to enrolment in the study. Samples were collected between 2001 and 2021, aliquoted and stored at −80°C. Only samples with a maximum of two freeze-thaw cycles were selected. Diagnosis of MS was according to the McDonald criteria.^1^ Diagnosis of NMOSD followed the international consensus criteria for NMOSD.^4^ Age, sex, diagnosis, disease-modifying therapy, EDSS, presence of OCBs, number of T2 lesions, number of Gd+lesions, relapse status, IgG index, AQP4 antibody status, CSF cell count and protein levels at the time of sample collection are reported in online supplemental table 1. All MS and NMOSD sample donors were untreated at the time of collection, except for three NMOSD cases who were treated with systemic corticosteroids. EDSS scores were available for 28 of 33 MS cases in the discovery cohort, and all 29 cases in the validation cohort. Based on the discovery cohort data, 34 samples in the validation cohort were estimated to provide 80% power to identify a correlation coefficient of 0.6 at p<0.0025, however, sample size was limited by the availability of samples from untreated MS cases.

### Lipid extraction

Lipids were extracted from 200 µL CSF with the single phase 1-butanol/methanol (BUME) 1:1 (v/v) method,^26^ by adding 650 µL of 1-butanol and 650 µL of methanol containing internal standards (table 1) and 0.01% (w/v) butylated hydroxytoluene (BHT). Samples were vortexed, sonicated for 2 hours at 4°C in a sonicating water bath and centrifuged at 16 000 g for 10 min. The supernatants were collected in glass vials and the pellets were re-extracted using 1:1 (v/v) BUME with 1-hour sonication. Extracted supernatants were combined, dried down in a Savant SpeedVac SC210 at 35°C overnight and reconstituted in 200 µL of 80% methanol/20% High Performance Liquid Chromatography (HPLC)-grade water with 0.1% formic acid, 10 mM ammonium formate and 0.01% BHT. These were centrifuged at 2000 g for 10 min to pellet debris, transferred to glass vials and stored at −80°C.

**Table 1:** Internal standards for lipidomics

Standard	Supplier	Cat. #	Amount per sample (nmoles)	Precursor ion (m/z)	Production (m/z)	Retention time (min)
PC(19:0/19:0)	Avanti Polar Lipids	850367P	5	818.7(M+H)^+^	184.1	13.5
SM(d18:1/17:0)	Cayman Chemical	25592	2	717.6(M+H)^+^	184.1	12.4
GluCer(d18:1/12:0)	Avanti Polar Lipids	860543	2 (discovery)	644.6(M+H)^+^	264.3	10.8
GluCer(d18:1/17:0)	Avanti Polar Lipids	860569P	2 (validation)	714.6(M+H)^+^	264.3	11.7
PS(17:0/17:0)	Avanti Polar Lipids	840028	2	762.5(M−H)^−^	269.2	11.7
PE(17:0/17:0)	Avanti Polar Lipids	830756	2	718.5(M−H)^−^720.6(M+H)^+^	269.2(M−H)^−^579.5(M+H)^+^	12.5
PG(17:0/17:0)	Avanti Polar Lipids	830456	2 (discovery)	749.5(M−H)^−^	269.2	11.6
CE(17:0)	Avanti Polar Lipids	700186	2 (discovery)5 (validation)	656.6(M+NH_4_)^+^	369.4	15.9
TG(17:0/17:0/17:0)	Cayman Chemical	19722	2 (discovery)	866.8(M+NH_4_)^+^	579.5	15.7
TG(16:0/16:0/16:0)-d31	Cayman Chemical	23334	5 (validation)	918.4(M+NH_4_)^+^	612.8	15.4
PA(17:0/17:0)	Avanti Polar Lipids	830856	1	675.5(M−H)^−^	269.2	12.4
PI(18:1/15:0)-d7	Avanti Polar Lipids	791641C	1	828.6(M−H)^−^	241.0	10.6
Cholesterol(18:1)-d7	Avanti Polar Lipids	791645	1 (discovery)5 (validation)	376.4(M+H−H_2_O)^+^	161.1	9.3
Cer(d18:1/17:0)	Avanti Polar Lipids	860517P	0.5	552.5(M+H)^+^	264.3	13.2
LacCer(d18:1/12:0)	Avanti Polar Lipids	860545P	0.5	806.6(M+H)^+^	264.3	10.6
ST(d18:1/17:0)	Avanti Polar Lipids	860572P	0.5	794.6(M+H)^+^	264.3	11.4
LPC(17:0)	Avanti Polar Lipids	855676	0.5	510.4(M+H)^+^	184.1	6.4
LPE(17:1)	Avanti Polar Lipids	856707	0.5	466.2(M+H)^+^	325.2	4.5
LPS(17:1)	Avanti Polar Lipids	858141	0.5 (discovery)	508.3(M−H)^−^	421.2	3.1
Sph(d17:1)	Avanti Polar Lipids	860640P	0.2	286.3(M+H)^+^	250.2	5.9
S1P(d17:1)	Cayman Chemical	22498	0.2	366.2(M+H)^+^	250.2	2.5
AcCa(16:0)-d3	Avanti Polar Lipids	55107	0.2 (discovery)	403.4(M+H)^+^	85.0	5.6
GM3(d18:1/18:0)-d5	Avanti Polar Lipids	860073	0.2 (discovery)	1185.8(M−H)^−^	290.1	11.7

AcCa, acyl carnitine; CE, cholesterol ester; Cer, ceramide; GluCer, glucosylceramide; GM3, ganglioside GM3LacCer, lactosylceramide (standard for dihexosylceramide); LPC, lysophosphatidylcholine; LPE, lysophosphatidylethanolamine; LPS, lysophosphatidylserine; PA, phosphatidic acid; PC, phosphatidylcholine; PE, phosphatidylethanolamine; PG, phosphatidylglycerol; PI, phosphatidylinositol; PS, phosphatidylserine; SM, sphingomyelin; S1P, sphingosine 1-phosphate; Sph, sphingosine; ST, sulfatide; TG, triglyceride

### Lipid quantification

Lipids were detected by multiple reaction monitoring on a TSQ Altis triple quadrupole mass spectrometer with Vanquish HPLC system. A 2.1×100 mm Waters Acquity CSH C18 column was used at a flow rate of 0.28 mL/min with a binary solvent system comprising mobile phase A: 60% acetonitrile/40% water/10 mM ammonium formate/0.1% formic acid; and B: 10% acetonitrile/90% isopropanol/10 mM ammonium formate/0.1% formic acid. Run time was 25 min: 0–3 min, 20% B; 3–5.5 min, ramp to 45% B; 5.5–8 min, ramp to 65% B; 8–13 min ramp to 85% B; 13–14 min, ramp to 100% B; 14–20 min, hold at 100% B; 20–20.5 min, decrease to 20% B; 20.5–25 min hold at 20% B^26^. Samples were randomised, with each run in positive and negative ion mode back-to-back. Every fifth injection was a solvent blank. Precursor and product ion pairs are listed in online supplemental table 2. Peaks were integrated using TraceFinder V.5.1 (Thermo Fisher Scientific). Quantifiable peaks comprised a minimum of eight consecutive scans with an area greater than 1000 units. Of the 161 lipids quantified, 28 had missing values in at least one sample. Only peaks present in at least 70% of samples were quantified. Lipid identifications were verified using retention time versus m/z plots and high-resolution untargeted lipidomic data collected on a Thermo Fisher Q-Exactive HF-X mass spectrometer using the same HPLC, column and solvents. The amount of each lipid was calculated relative to its class-specific internal standard. A Lipidomics Standards Initiative reporting checklist has been published: https://doi.org/10.5281/zenodo.10995469.

### Multiplex immunoassay

A custom ProcartaPlex assay (Thermo Fisher Scientific) was used to measure levels of 15 pro-inflammatory cytokines and chemokines (migration inhibitory factor (MIF), tumour necrosis factor (TNF)-α, interferon-γ, CXCL10, CXCL13, interleukin (IL)-1β, IL-6, IL-8, IL-12/IL-23p40, IL-15, IL-17A, IL-22, Chemokine (C-C motif) ligand 5 (CCL5), TNF-related apoptosis-inducing ligand (TRAIL) and soluble triggering receptor expressed on myeloid cells 2 (sTREM2)) in 74 CSF samples from the discovery cohort (9 NIND, 22 OIND, 32 MS, 11 NMOSD). Results are the mean of duplicate measurements for each sample. The assay was performed by the Bursky Center for Human Immunology & Immunotherapy at Washington University, Missouri, USA.

### Statistical analysis

R studio (V.2023.09.1) was used for statistical analyses. Lipids that were not detected in particular samples were assigned a value of 1/5 of the minimum value for that species for statistical analyses. Lipid concentrations and clinical covariates were log_10_-transformed to improve normality, which was assessed with the Anderson-Darling test. Lipid levels were compared between categorical variables (disease, EDSS) using one-way analysis of variance (ANOVA) adjusted for age and sex, followed by Tukey’s multiple comparisons post hoc test. Correlations were tested using Pearson’s analysis. Partial correlation was used to account for the effect of age. Gd+lesions were categorised into a binary outcome based on the presence or absence of lesions. P values arising from ANOVA or correlation analyses were corrected for false discovery rate (FDR) using the Benjamini-Hochberg method, with q<0.05 considered significant.

Random forest models were performed with the ClassifyR, dplyr, devtools, S4Vectors and ranger packages in R. Feature selection was confirmed using random forest biomarker analysis in MetaboAnalyst V.5.0, where sensitivity and specificity values were derived from confusion matrices. MetaboAnalyst V.5.0 was used for partial-least squares discriminant analyses (PLS-DA) and to generate Variable Importance in Projection plots. Bar, scatter and volcano plots were created using GraphPad Prism (V.9.5.1). Heatmaps were created using MetaboAnalyst V.5.0 and SRplot.

## Results

### CSF sphingolipids are increased in NMOSD relative to MS

To establish a targeted assay for CSF lipids, 353 lipid species were screened in a pooled CSF sample, resulting in 161 quantifiable lipids (online supplemental table 2). The average lipid composition was similar to that of previous studies,^27^ with cholesterol accounting for 67% of CSF lipid, followed by triglycerides (TGs) at 17% and cholesterol esters (CEs) at 11%. These 161 lipids were quantified in CSF samples from 33 MS, 28 NIND, 27 OIND and 11 NMOSD cases (discovery cohort) (table 2). Age (*F*(3, 95)=1.8, p*=*0.2, one-way ANOVA) and sex (χ^2^(3)=6.8, p*=*0.08, χ^2^ test) did not differ significantly between the groups. No lipids differed significantly in abundance between men and women, whereas 10 lipids were significantly correlated with age (table 3).

**Table 2:** Cohort characteristics

Sample group	n	Age (years; mean±SD)	Sex (% female)	CSF cells (per mL; mean±SD)	CSF protein (mg/dL; mean±SD)	Neurological disorder (n)
NIND (discovery)	28	45±18	75	1±2	38±23	Normal control (1), headache/migraine (7), pseudotumour cerebri (7), NPH (4), subjective memory issues/cognitive decline (2), syringomyelia (1), gait disturbances (1), CVST (1), PSP (1), tectal glioma (1), papilloedema (1), anti-epileptic drug toxicity (1).
OIND (discovery)	27	40±16	59	15±40	56±38	Neurosarcoidosis (4), MOGAD (4) meningitis (3), transverse myelitis (3), CIDP (3), ADEM (2), CNS lymphoma (2), CNS vasculitis (2), small fibre neuropathy (1), AMAN (1), mononeuritis multiplex (1), CLIPPERS (1).
NMOSD (discovery)	11	41±17	100	114±226*	112±176†	In relapse (8), in remission (3).AQP-4 seronegative (5), seropositive (6).
MS (discovery)	33	37±11	67	13±26	34±11	RRMS (33): in relapse (13), in remission (20).
MS (validation)	29	38±12	64	8±10	38±12	RRMS (27): in relapse (8), in remission (19).PPMS (2): in relapse (1), in remission (1).

All samples were from people who were untreated at the time of lumbar puncture except for three NMOSD cases that had steroid treatment.

* Significantly higher in NMOSD compared tocompared with all other groups (*F*(3, 52)=6.4, pp*=*0.0009).

† Significantly higher in NMOSD compared with MS and NIND (*F*(3, 85)=6.5, p*=*0.0005).

ADEMacute disseminated encephalomyelitisAMANacute motor axonal neuropathyCIDPchronic inflammatory demyelinating polyneuropathyCLIPPERSchronic lymphocytic inflammation with pontine perivascular enhancement responsive to steroidsCNScentral nervous systemCSFcerebrospinal fluidCVSTcerebral venous sinus thrombosisMOGADmyelin oligodendrocyte glycoprotein antibody diseaseMSmultiple sclerosisNINDnon-inflammatory neurological diseases NMOSDneuromyelitis optica spectrum disorderNPHnormal pressure hydrocephalusOINDother inflammatory neurological diseases PPMSprimary progressive MSPSPprogressive supranuclear palsyRRMSrelapsing-remitting MS

**Table 3:** Cerebrospinal fluid lipids correlated with age

Lipid	Discovery cohort			Validation cohort		
	r	P value	q value	r	P value	q value
SM(d18:2/20:0)	**0.34**	**0.0005**	**0.044**	**0.55**	**0.0018**	**0.006**
ST(d18:2/24:0)	0.34	0.0006	0.044
SM(d18:2/22:0)	**0.31**	**0.0017**	**0.047**	**0.50**	**0.0056**	**0.098**
ST(d18:2/18:0)	0.30	0.0024	0.047
LPC(22:5)	0.30	0.0025	0.047	0.38	0.044	0.051
HexCer(d18:1/22:0-OH)	0.30	0.0026	0.047	0.20	0.39	0.39
SM(d18:1/24:1)	**0.30**	**0.0027**	**0.047**	**0.51**	**0.0050**	**0.098**
PI(18:0/18:1)	0.30	0.0028	0.047
SM(d18:1/22:0)	**0.30**	**0.0029**	**0.047**	**0.43**	**0.020**	**0.028**
SM(d18:2/18:0)	**0.30**	**0.0029**	**0.047**	**0.61**	**0.0004**	**0.003**

Correlation coefficient (r), p and q values for lipids that were significantly correlated with age in the discovery cohort (q*<*0.05). These correlations were tested in the validation cohort samples, resulting in significant correlations for five SM species (bold font).

HexCerhexosylceramideLPClysophosphatidylcholinePIphosphatidylinositolSMsphingomyelinSTsulfatide

**Figure 1:**
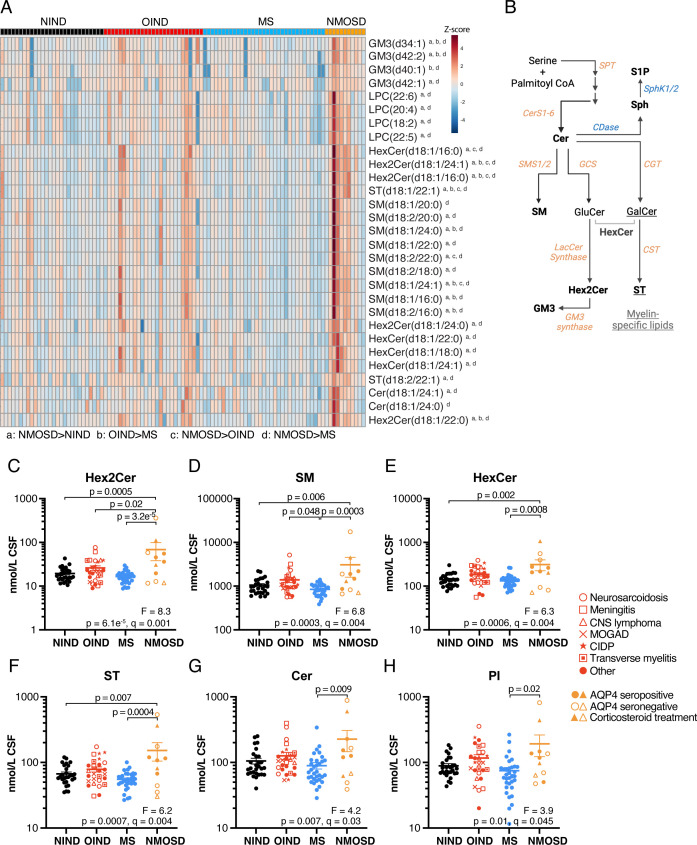
Sphingolipid levels are higher in CSF of NMOSD compared with MS cases. (**A**) Heatmap of lipids differing significantly between NIND, OIND, MS and NMOSD sample groups in one-way ANOVA adjusted for age and sex and corrected for FDR (q*<*0.05). Significant Tukey’s post hoc test comparisons (p*<*0.05) are denoted by superscript a: NMOSD>NIND, b: OIND>MS, c: NMOSD>OIND, d: NMOSD>MS. Heat map uses Euclidean distance measure with Ward clustering. (**B**) Biosynthetic pathway illustrating metabolic relationships between the sphingolipids. Anabolic enzymes are in orange while catabolic enzymes are in blue. (**C–H**) Lipid class totals whose levels differed significantly between the sample groups. ANOVA results are shown at the bottom of each graph and significant differences arising from Tukey’s post test are shown. The dashed lines and error bars show mean±SEM for each group. ANOVA, analysis of variance; AQP4, aquaporin-4; CDase, ceramidase; Cer, ceramide; CerS, ceramide synthase; CGT, ceramide galactosyltransferase; CIDP, chronic inflammatory demyelinating polyneuropathy; CNS, central nervous system; CSF, cerebrospinal fluid; CST, cerebroside sulfotransferase; FDR, false discovery rate; GalCer, galactosylceramide; GCS, glucosylceramide synthase; GluCer, glucosylceramide; GM3, ganglioside; HexCer, hexosylceramide; Hex2Cer, dihexosylceramide; MOGAD, myelin oligodendrocyte glycoprotein antibody disease; MS, multiple sclerosis; NIND, non-inflammatory neurological diseases; NMOSD, neuromyelitis optica spectrum disorder; OIND, other inflammatory neurological diseases; S1P, sphingosine-1-phosphate; SM, sphingomyelin; SMS, sphingomyelin synthase; Sph, sphingosine; SphK, sphingosine kinase; SPT, serine palmitoyltransferase; ST, sulfatide.

After correcting for FDR, 25 sphingolipids (9 SM, 4 ganglioside (GM3), 4 HexCer, 4 dihexosylceramide (Hex2Cer), 2 ST and 2 Cer) and 4 polyunsaturated LPCs differed significantly in abundance between the sample groups in one-way ANOVA adjusted for age and sex (figure 1A,B, online supplemental table 3A). Mean levels of all 29 lipids were significantly higher in NMOSD compared with MS (p*<*0.05, Tukey’s post test), 26 were higher in NMOSD compared with NIND and 6 were higher in NMOSD compared with OIND. Eleven sphingolipids were also significantly higher in OIND compared with MS. Levels of these lipids were particularly high in a subset of OIND cases including viral and lymphomatous meningitis, CNS lymphoma, neurosarcoidosis and chronic inflammatory demyelinating polyneuropathy (CIDP) (online supplemental figure 1). Comparing MS cases experiencing a relapse (n*=*12) to those in remission (n*=*21), 35 lipids were significantly lower during relapse in univariate t-tests, however, these associations did not survive FDR correction (online supplemental table 4).

When analysed as lipid class totals (online supplemental table 3B), total Hex2Cer was higher in NMOSD compared with all other groups (figure 1C). Total SM was higher in NMOSD compared with NIND and MS, and higher in OIND compared with MS (figure 1D). Total HexCer (figure 1E) and ST (figure 1F) were increased in NMOSD compared with NIND and MS; and total Cer (figure 1G) and phosphatidylinositol (figure 1H) were higher in NMOSD compared with MS only.

**Figure 2:**
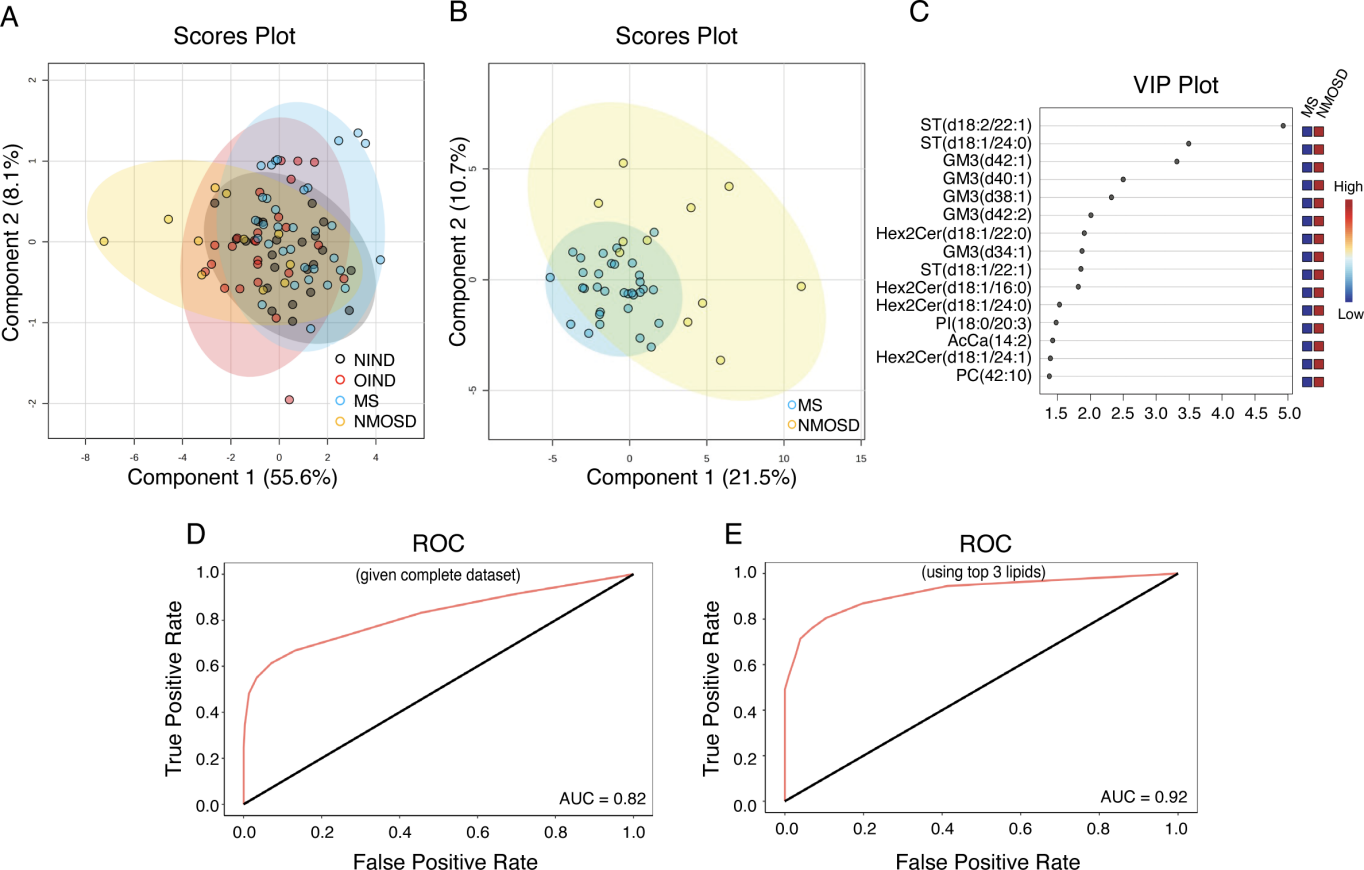
CSF sphingolipid levels can differentiate NMOSD from MS. (**A**) PLS-DA scores plot for clustering of NIND (grey), OIND (red), MS (blue) and NMOSD (yellow) based on CSF lipids. (**B**) PLS-DA and (**C**) VIP scores plot of MS (blue) and NMOSD (yellow) samples only. (**D&E**) ROC plots from random forest models for the differentiation of NMOSD from MS using (**D**) all lipids, or (**E**) only the top three performing lipids GM(d42:2), Hex2Cer(d18:1/22:0) and GM3(d38:1). AcCa, acyl carnitine; AUC, area under the curve; CSF, cerebrospinal fluid; GM3, ganglioside; Hex2Cer, dihexosylceramide; MS, multiple sclerosis; NIND, non-inflammatory neurological diseases; NMOSD, neuromyelitis optica spectrum disorder; OIND, other inflammatory neurological diseases; PC, phosphatidylcholine; PI, phosphatidylinositol; PLS-DA, partial-least squares discriminant analyses; ROC, receiver operating characteristic; ST, sulfatide; VIP, Variable Importance in Projection.

The four disease groups could not be differentiated with PLS-DA (figure 2A) or random forest analysis. Differentiation of NMOSD from MS with PLS-DA was better (figure 2B), driven by ST, GM3 and Hex2Cer species (figure 2C). Random forest analysis including all lipids produced an area under the curve (AUC) of 0.82 for differentiating NMOSD from MS (figure 2D, online supplemental table 5). Using only the three most influential lipids in the model—GM3(d42:2), Hex2Cer(d18:1/22:0) and GM3(d38:1), the AUC improved to 0.92 (figure 2E), with 91% sensitivity and 91% specificity. Hex2Cer(d18:1/22:0) alone differentiated NMOSD from MS with 73% sensitivity and 100% specificity based on a threshold of 1.1 nmol/L CSF.

### Sphingolipids and LPCs are correlated with inflammatory mediators and total CSF protein

**Figure 3:**
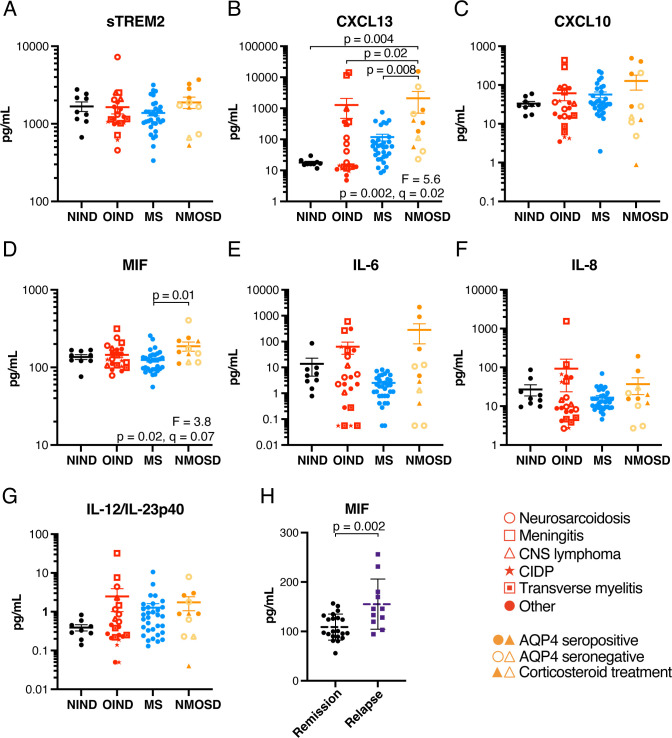
CXCL13 levels are higher in NMOSD. (**A–G**) sTREM2, cytokine and chemokine levels in 9 NIND, 22 OIND, 32 MS and 11 NMOSD CSF samples. Significant analysis of variance results are shown underneath the data points (**B and D**) and significant differences arising from Tukey’s post test are shown above the data points. Dashed line and error bars show mean±SEM for each group. (**H**) MIF levels in MS cases in remission compared with those experiencing a relapse. AQP4, aquaporin-4; CIDP, chronic inflammatory demyelinating polyneuropathy; CNS, central nervous system; CSF, cerebrospinal fluid; CXCL13, C-X-C motif chemokine ligand 13; IL, interleukin; MIF, migration inhibitory factor; MS, multiple sclerosis; NIND, non-inflammatory neurological diseases; NMOSD, neuromyelitis optica spectrum disorder; OIND, other inflammatory neurological diseases; sTREM2, soluble triggering receptor expressed on myeloid cells 2.

Given that sphingolipid and LPC species were higher in a subset of NMOSD and OIND cases, we aimed to determine if levels of these lipids correlated with inflammatory cytokines and chemokines. Of 15 analytes measured by multiplex immunoassay, 7 were within the quantifiable range (figure 3A–G, online supplemental table 6). CXCL13 levels were significantly higher in NMOSD (median level of 340.9 pg/mL) compared with NIND (16.1 pg/mL), OIND (14.6 pg/mL) and MS (58.4 pg/mL) (figure 3B), and trended higher in MS cases compared with NIND (not significant). MIF (figure 3D) was significantly higher in NMOSD compared with MS in univariate ANOVA, but not after correcting for FDR. MIF was also higher in relapsing MS cases compared with those in remission (t*=*3.3, p*=*0.002, q*=*0.01) (figure 3H).

**Figure 4:**
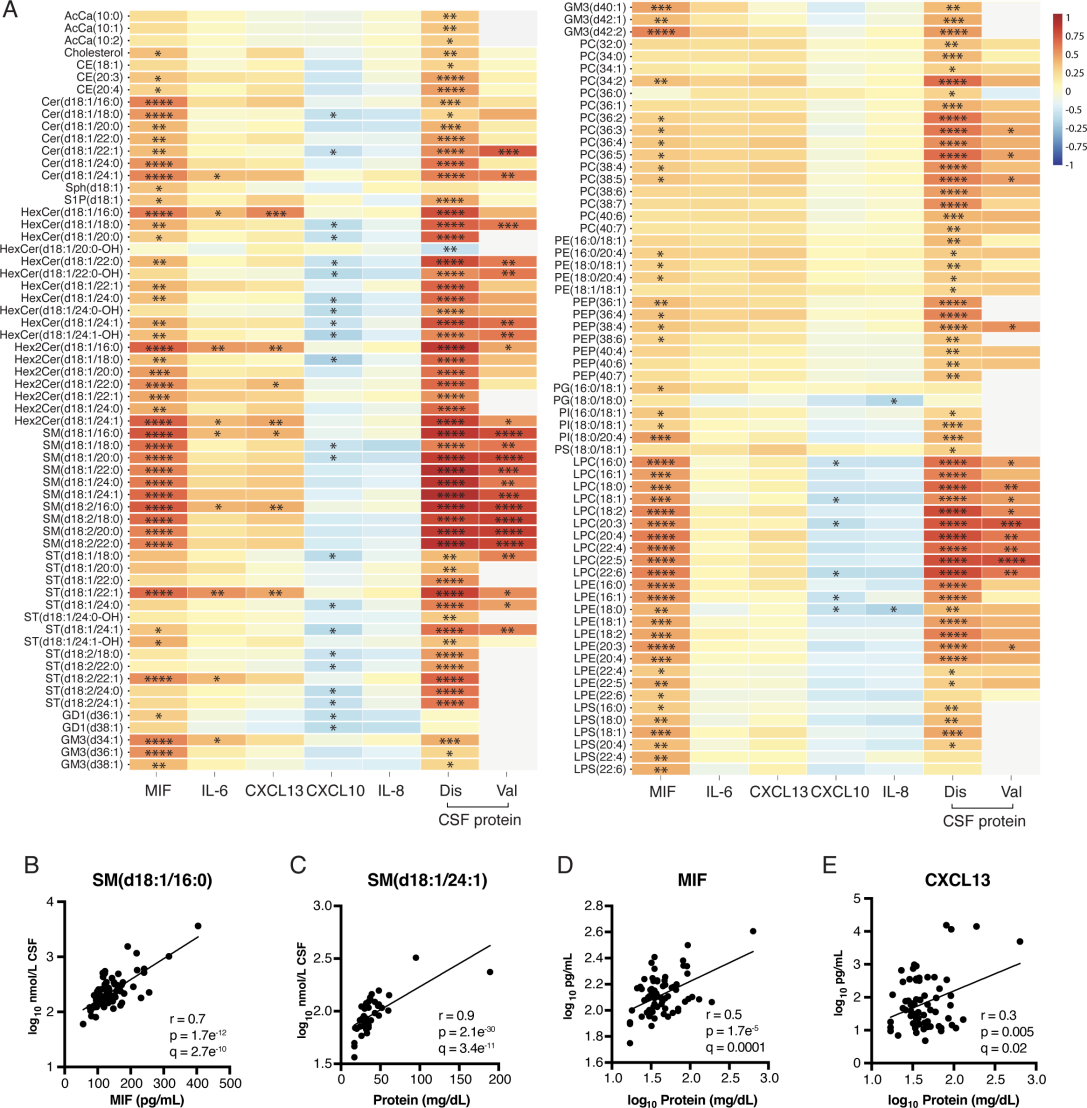
CSF sphingolipids and lysophospholipids are correlated with MIF and CSF protein. (**A**) Heatmap showing CSF lipids that were significantly correlated with MIF, IL-6, CXCL13, CXCL10, IL-8, or CSF protein in the discovery (Dis) and validation (Val) cohorts: *q*<*0.05, **q*<*0.01, ***q*<*0.001, ****q*<*0.0001, no asterisk indicates non-significant (q*>*0.05). The colour scale shows the correlation coefficient (r). Grey signifies lipids that were not measured in the validation cohort. CSF protein values were available for 32 MS, 11 NMOSD, 24 OIND and 22 NIND samples in the discovery cohort and all 29 MS samples in the validation cohort. (**B–C**) The most significantly correlated lipids (lowest q value) to (**B**) MIF and (**C**) CSF protein in the discovery cohort. (**D–E**) Correlations between total CSF protein and (**D**) MIF and (**E**) CXCL13. Lines of best fit are shown. AcCa, acyl carnitine; CE, cholesterol ester; Cer, ceramide; CSF, cerebrospinal fluid; CXCL13, C-X-C motif chemokine ligand 13; GD, ganglioside GD1; GM3, ganglioside GM3; HexCer, hexosylceramide; Hex2Cer, dihexosylceramide; IL, interleukin; LPC, lysophosphatidylcholine; LPE, lysophosphatidylethanolamine; LPS, lysophosphatidylserine; MIF, migration inhibitory factor; MS, multiple sclerosis; NIND, non-inflammatory neurological diseases; NMOSD, neuromyelitis optica spectrum disorder; OIND, other inflammatory neurological diseases; PC, phosphatidylcholine; PE, phosphatidylethanolamine; PEP, phosphatidylethanolamine plasmalogen; PG, phosphatidylglycerine; PI, phosphatidylinositol; PS, phosphatidylserine; SM, sphingomyelin; ST, sulfatide.

Remarkably, 94 lipids were significantly correlated with MIF levels after FDR correction, the most strongly correlated being SMs, Hex2Cers and LPCs (figure 4A,B, online supplemental table 7). Nine sphingolipids were positively correlated with IL-6, and seven with CXCL13. In contrast, 28 sphingolipids and lysophospholipids were inversely correlated with CXCL10. Overall, the lipids that were higher in the NMOSD and OIND sample groups were positively correlated with MIF, IL-6 and CXCL13, and inversely correlated with CXCL10.

Total CSF protein was significantly higher in NMOSD compared with MS and NIND (table 2). After correcting for FDR, 117 lipids were positively correlated with CSF protein, of which 55 were sphingolipids (figure 4A and C, online supplemental table 8A). These lipids overlapped substantially with those correlated to inflammatory mediators. From the cytokines profiled, MIF and CXCL13 were positively correlated with CSF protein (figure 4D–E, online supplemental table 9).

### CXCL10, CXCL13 and IL-12/IL-23p40 are associated with IgG index

No lipids were correlated with IgG index in MS cases, however, heterodimeric IL-12 and IL-23 sharing the same p40 subunit (IL-12/IL-23p40) (r*=*0.5, p*=*0.001, q*=*0.009), CXCL13 (r*=*0.5, p*=*0.005, q*=*0.01) and CXCL10 (r*=*0.5, p*=*0.006, q*=*0.01) were positively correlated with IgG index (online supplemental table 9).

### Association of CSF lipids with the presence of Gd+ lesions

**Figure 5:**
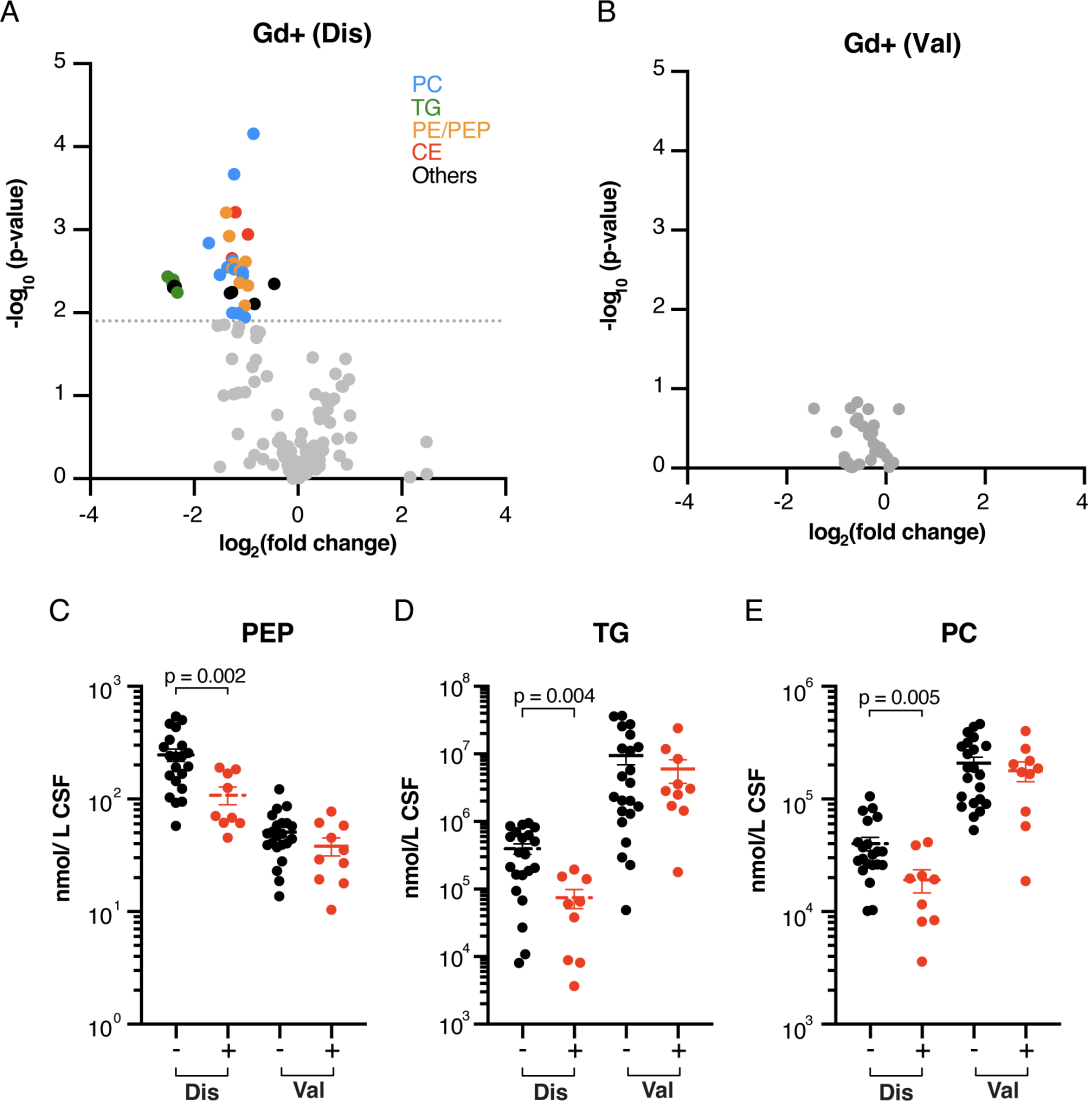
CSF lipids were not reproducibly associated with the presence of Gd+lesions in MS. (**A**) Volcano plot shows that 39 lipids were significantly reduced in CSF of MS cases from the discovery cohort with Gd+lesions (n*=*9), compared to with those without (n*=*21). The grey dotted line indicates q*<*0.05 threshold. (**B**) The same 39 lipids were unaffected by the presence of Gd+lesions in the validation cohort (n*=*10 Gd+, n*=*21 Gd−). (**C–E**) Lipid class totals for (**C**) PEP, (**D**) TG and (**E**) PC were significantly lower in MS cases with Gd+lesions (+) in the discovery cohort (Dis) but not in the validation cohort (Val). CE, cholesterol ester; CSF, cerebrospinal fluid; Gd, gadolinium; MS, multiple sclerosis; PC, phosphatidylcholine; PE, phosphatidylethanolamine; PEP, phosphatidylethanolamine plasmalogen; TG, triglyceride.

Gd+lesions are active inflammatory lesions with local blood-brain barrier disruption.^6^ Thirty-nine lipids were significantly reduced in the CSF of MS cases with Gd+lesions (n*=*9) compared with those without (n*=*21) (figure 5A, online supplemental table 10). These consisted primarily of phosphatidylcholine (PC), TG, CE, phosphatidylethanolamine and phosphatidylethanolamine plasmalogen (PEP) species. When analysed as lipid class totals, PEP (t*=*3.4, p*=*0.002, q*=*0.04), TG (t*=*3.1, p*=*0.004, q*=*0.04) and PC (t*=*3.1, p*=*0.005, q*=*0.04) were significantly reduced in the CSF of MS cases with Gd+lesions (figure 5C–E).

### Cholesterol esters are inversely correlated with EDSS scores

**Figure 6:**
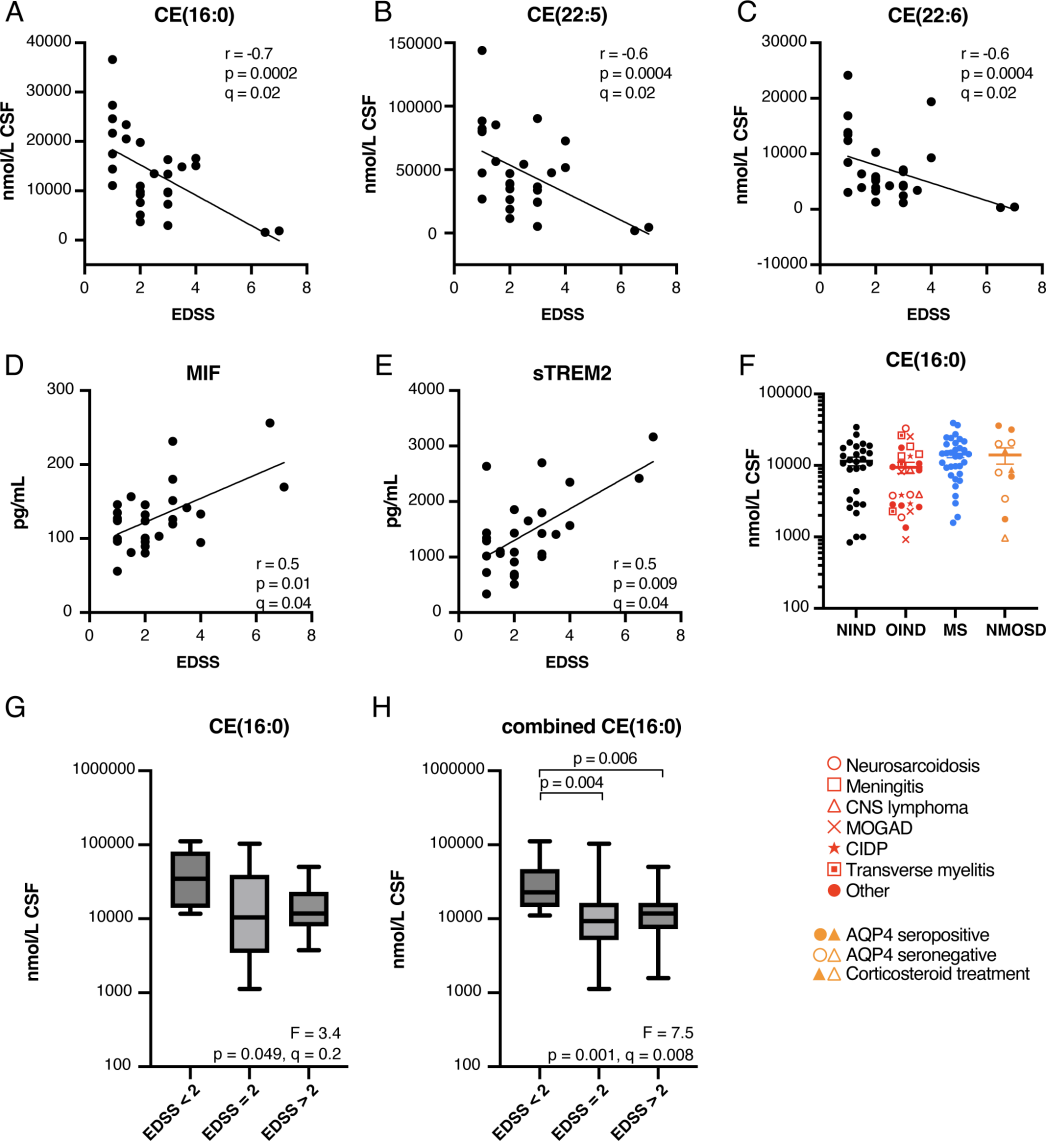
CE(16:0) is inversely correlated with EDSS. (**A–E**) Correlations between EDSS score and (**A**) CE(16:0), (**B**) CE(22:5), (**C**) CE(22:6), (**D**) MIF and (**E**) sTREM2 in the discovery cohort (n*=*28). (**F**) Levels of CE(16:0) across the four sample groups in the discovery cohort. (**G–H**) CE(16:0) in MS cases from the (**G**) validation cohort (n*=*29), and (**H**) combined discovery and validation cohorts, categorised according to EDSS score. Box and whiskers show the IQR and range, respectively; the horizontal line shows the mean. AQP4, aquaporin-4; CE, cholesterol ester; CIDP, chronic inflammatory demyelinating polyneuropathy; CNS, central nervous system; CSF, cerebrospinal fluid; EDSS, Expanded Disability Status Scale; MIF, migration inhibitory factor; MOGAD, myelin oligodendrocyte glycoprotein antibody disease; MS, multiple sclerosis; NIND, non-inflammatory neurological diseases; NMOSD, neuromyelitis optica spectrum disorder; OIND, other inflammatory neurological diseases; sTREM2, soluble triggering receptor expressed on myeloid cells 2.

Three CEs (CE(16:0), CE(22:5) and CE(22:6)) were inversely correlated with EDSS scores after correcting for FDR (figure 6A–C, online supplemental table 11). Total CE (r*=*−0.5, p*=*0.009, q*=*0.2) and total cholesterol (r*=*0.3, p*=*0.2, q*=*0.3) were not correlated with EDSS. From the cytokines, MIF and sTREM2 were significantly correlated with EDSS (figure 6D–E).

Despite the inverse correlation with EDSS, mean levels of CE(16:0) (figure 6F) and other CE species did not differ between the four sample groups. CE(16:0) trended lower in MS cases undergoing relapse, however, this was not statistically significant after FDR correction (t*=*2.1, p*=*0.04, q*=*0.2).

### Validation of the inverse association between CSF CE(16:0) and EDSS scores

Since there have been few prior studies looking at associations between CSF lipids and clinical measures of disease activity in MS, we sought to validate the associations between CSF lipids and EDSS, total CSF protein and Gd+lesions in an independent cohort of 29 MS samples. There were no significant differences in age (t*=*0.3, p*=*0.7) and sex (χ^2^(1)=0.8, p*=*0.4) between the two MS sample cohorts, although the discovery cohort included a higher proportion of MS cases in relapse (^2^χ^2^(1)=5.6, p*=*0.02). Five SM species remained significantly correlated with age in the validation cohort (table 3) and 37 lipids were significantly associated with CSF protein (figure 4A and online supplemental table 8B), all of which were in common with the discovery cohort. We were not able to reproduce significant differences in lipid or lipid class totals between MS cases with or without Gd+lesions in the validation cohort (figure 5B–E), and no lipids were significantly affected by relapse status.

Due to the limited range of EDSS scores in our validation cohort, associations between CE levels and EDSS were tested by categorising the samples into three groups based on EDSS: <2, =2, or >2. CE(16:0), but not CE(22:5) or CE(22:6), varied significantly with EDSS in one-way ANOVA adjusted for age and sex (figure 6G). However, this association and the post tests were not significant after FDR correction, potentially due to the limited range of EDSS scores. On combining the two MS sample cohorts CE(16:0) was significantly higher in cases with EDSS<2 (figure 6H), whereas CE(22:5) (*F*(2, 52)=5, p*=*0.01, q*=*0.052) and CE(22:6) (*F*(2, 52)=2.8, p*=*0.07, q=0.1) were not.

## Discussion

This study establishes that levels of multiple sphingolipid and LPC species are significantly higher in the CSF of NMOSD and other neuroinflammatory diseases (meningitis, CNS lymphoma, neurosarcoidosis, CIDP) compared with MS and NIND. Over half of the lipids measured in CSF were correlated with levels of the inflammatory cytokine MIF, raising the possibility of a functional interaction in which MIF regulates CSF sphingolipid and lysophospholipid levels and 55/62 measured sphingolipids were positively correlated with CSF protein. We demonstrate that levels of CE(16:0) in the CSF are inversely associated with EDSS in two independent MS sample cohorts, whereas no lipids were reproducibly associated with the presence of Gd+lesions. We also confirm prior findings that MIF^28 29^ and sTREM2^30^ are positively correlated with EDSS.

Myelin is enriched in the sphingolipids SM, HexCer and ST^17^ that were higher in NMOSD and OIND cases, suggesting that their increased levels in CSF could result from myelin degradation. In support of this, SM levels are elevated in the CSF of people with peripheral demyelinating neuropathies compared with those with non-demyelinating disorders or neuroinflammatory conditions with blood-brain barrier dysfunction.^31^ Compared with MS, NMOSD features larger spinal cord and periventricular lesions that could result in greater release of myelin lipids into the CSF. Similarly, cranial and spinal cord lesions on MRI are common in neurosarcoidosis,^32^ while CIDP is characterised by peripheral nerve demyelination.^33^ Although meningitis or lymphoma are not primary demyelinating conditions, acute inflammation and tissue atrophy in these conditions can result in significant myelin loss.^34^ In this study, we did not observe any significant associations between lipid levels and MRI lesion load in people with MS, however, we did not test associations between CSF lipid levels and lesion size or location in NMOSD. Future studies should determine if CSF sphingolipid levels are correlated with MRI proxies of myelin loss, such as total white matter volume, white matter lesion volume and the location and size of lesions in both NMOSD and MS.

Prior studies have shown higher CSF ceramides and HexCers in MS relative to other neurological diseases.^19 21 22^ C16 and C24 ceramide loading induce mitochondrial dysfunction in cultured neurons,^19^ while C16 and C24:1 HexCer were positively correlated with EDSS (at univariate p<0.05) and higher in MS cases with >9 MRI lesions.^22^ These findings were not replicated in our study, possibly because HexCer levels are elevated in progressive but not relapsing-remitting MS (RRMS).^22^ Our results concur with a prior study demonstrating lower SM levels in MS compared with other neurological diseases.^24^ Total SM levels were also decreased in the plasma of people with MS compared with those with other neurological diseases,^35^ indicating possible correlations between CSF and plasma lipid biomarkers.

The four LPCs that were higher in NMOSD compared with MS and NIND had polyunsaturated (18:2, 20:4, 22:5, 22:6) rather than the more abundant saturated or monounsaturated (16:0, 18:0, 18:1) fatty acids. These polyunsaturated fatty acids are more common at the sn2 position of PC, whereas saturated fatty acids are more common at sn1,^36^ suggesting that increased LPCs in NMOSD CSF could be derived from phospholipase A1 (PLA_1_)-mediated cleavage of PC. The involvement of PLA_1_ isoforms in inflammatory responses is much less well studied than for PLA_2_. Treatment of astrocytes with serum from NMOSD cases upregulates secreted PLA_1_A along with various inflammatory chemokines,^37^ however, PLA_1_A is selective for phosphatidylserine and not thought to hydrolyse PC. Alternatively, increased levels of polyunsaturated LPC species could reflect CNS import through LPC transporters such as Mfsd2a,^38^ potentially as a mechanism to increase brain lipid synthesis.^39^

The possibility that increased sphingolipid and LPC levels in CSF result from demyelination is supported by their strong correlation with MIF, which is highly expressed in active MS lesions and acts as a chemoattractant for monocytes and microglia that mediate myelin break-down.^40^ MIF is required for sustained inflammatory activation of both innate and adaptive immune cells in experimental autoimmune encephalitis (EAE), a rodent model of autoimmune demyelination.^40 41^ We observed higher MIF levels in NMOSD compared with MS, in agreement with a prior study comparing conventional MS with the optic-spinal variant, which is now thought to be synonymous with NMOSD.^28^

An alternative explanation for increased sphingolipid levels in the CSF of NMOSD and OIND cases is local biosynthesis, since multiple metabolically-related sphingolipids were increased (figure 1C). Ceramide synthesis is increased in astrocytes within MS lesions or during cuprizone-induced demyelination and, in combination with TNF-α, induces oligodendrocyte apoptosis in astrocyte-oligodendrocyte co-cultures.^42^ Lactosylceramide (Hex2Cer) synthesis by astrocytes is necessary for the production of pro-inflammatory chemokines and cytokines, recruitment of microglia and monocytes and demyelination in EAE.^43^ In our study, Hex2Cer species were among the most highly increased lipids in NMOSD and OIND, raising the possibility that the synthesis of these sphingolipids in astrocytes or other glia drives inflammation. Supporting this, CSF levels of the astrocyte marker glial fibrillar acidic protein (GFAP) are higher in NMOSD relative to MS,^44 45^ and strongly affected by the presence^45^ and size^44^ of spinal cord lesions. In contrast, myelin basic protein levels are not significantly higher in the CSF of NMOSD compared with MS,^44 46^ suggesting that altered lipid levels may be related to astrocyte pathology rather than myelin loss. Furthermore, plasma C14 and C16 ceramides were positively correlated with EDSS scores and NfL levels in a recent study with Korean patients with NMOSD.^47^ Unlike NfL and GFAP, ceramide levels were not associated with relapse status and did not differ between NMOSD and healthy controls,^47^ suggesting that their levels are related to clinical disability rather than tissue destruction.

A third possibility for increased lipid levels in CSF is increased transfer across the blood-CSF barrier. In that case, one would expect the most abundant lipids in plasma—TGs, CEs and PCs—to correlate most strongly with CSF protein, whereas we observed the strongest correlations to sphingolipids and lysophospholipids. Furthermore, in a recent study, 90% of CSF lipids were correlated with total CSF protein in healthy control subjects, in whom the blood-CSF barrier is presumably intact.^27^ Since CSF protein levels are higher in people with severe neuroinflammatory conditions compared with those with non-inflammatory disorders or MS,^21 48^ high CSF sphingolipid and LPC levels probably reflect increased production in the CNS related to the severity of CNS inflammation and/or demyelination.

Both CXCL13 and CXCL10 were correlated with IgG index in MS cases, in agreement with prior studies.^9 10^ CXCL13 recruits B cells to sites of inflammation through their CXCR5 receptors, whereas CXCL10 is thought to fuel MS pathogenesis through type 1 T helper and natural killer cell recruitment.^49^ CXCL10 was inversely correlated with HexCer, SM and ST species with N-acyl chains that are enriched in neuronal membranes (C18-C20) and myelin (C22-C24), whereas CXCL13 and IL-6 were positively correlated with HexCer and SM containing C16 N-acyl chains that are more abundant outside the CNS and probably not derived from neurons or myelin. This suggests that CXCL10 responses may counteract the processes that generate high lipid levels in NMOSD and other neuroinflammatory disorders. Our observation of higher CXCL13 levels in NMOSD compared with MS and NIND agrees with prior studies,^9 50^ whereas we did not replicate previous reports that CXCL13 is positively correlated with EDSS in RRMS.^9 10 50^

A novel and important finding was the inverse association of CE(16:0) with EDSS in both MS cohorts. While cholesterol accounts for 40% of myelin lipid,^17^ CEs comprise only 0.1–0.2% of total CNS cholesterol in healthy adults.^51^ CEs accumulate in lipid droplets within MS lesions as microglia and macrophages phagocytose and esterify cholesterol released from myelin.^52 53^ Reduced CSF CE(16:0) levels with increasing EDSS might therefore be related to reduced CE efflux from increasingly dysfunctional, cholesterol-engorged phagocytes. Decreased CE hydrolase activity in MS may exacerbate this CE storage,^54^ although in our study CE levels did not differ between the MS, NIND, OIND or NMOSD groups. Alternatively, a greater proportion of CEs may cross from the CSF into the blood of people with higher disability scores. Cholesterol must be hydroxylated (forming oxysterols) for export from the CNS, and a prior study reported that serum oxysterols are correlated with cumulative T2 lesion number.^55^ Prior studies have also established that total serum cholesterol and low density lipoprotein cholesterol are positively correlated with disability scores and T2 lesion number.^55 56^ Future studies should determine if CSF CE(16:0) is a useful predictor of MS disease progression and therapeutic response.

The gold standard diagnostic marker for NMOSD is serum AQP4 antibodies. Since 20–40% of NMOSD cases are negative for AQP4 antibodies^5^ and treatment of NMOSD with some MS therapeutics can be harmful,^3^ additional biomarkers for distinguishing NMOSD from MS could aid diagnosis and treatment. Among other markers, current studies are investigating GFAP^44^ and granulocyte activation markers.^57^ We present initial evidence that CSF sphingolipids provide a high degree of sensitivity and specificity for separating NMOSD from MS, however, an important limitation of our study was the low NMOSD sample size, as it is a rare disease. This prevented us from comparing lipid levels between AQP4 antibody-positive and AQP4 antibody-negative NMOSD, and we were unable to validate our findings on the basis of prior studies since this is the first study to compare the CSF lipidome between NMOSD and MS. Since our discovery cohort did not include any progressive MS cases, we cannot determine whether lipid levels are higher in NMOSD and OIND relative to all MS, or just RRMS. Our results therefore await validation in larger cohorts, and further investigation is needed to determine how well CSF lipids differentiate NMOSD from progressive MS and other neuroinflammatory, demyelinating diseases such as myelin oligodendrocyte glycoprotein antibody-associated disease.

Another limitation of our study was the absence of healthy controls, which prevented us from establishing the normal range for CSF lipids. CSF examination is not routinely carried out in healthy individuals and accordingly, NIND is commonly used as a control group for CSF biomarker studies.^21 28^ A third limitation is that the cohort of untreated MS cases used to validate associations of lipids with EDSS scores had a narrow spread of EDSS scores in the low range. This likely produced an underestimation of the strength of correlations between lipid levels and EDSS. Lastly, BMI was not available for many of our samples, which prevented us from adjusting for BMI in our analyses. However, BMI did not correlate with any CSF lipids in the 33 samples for which this metric was available.

## Conclusions

In conclusion, this study has established that levels of multiple sphingolipid species are higher in the CSF of NMOSD and other neuroinflammatory disorders compared with MS, and demonstrated that levels of these lipids are strongly correlated with MIF and CSF protein levels. We established an inverse correlation between CE(16:0) and EDSS scores, and confirmed that MIF and sTREM2 are positively associated with EDSS. Fluid biomarkers that are indicative of disease progression and failed therapeutic response in MS would complement MRI analysis of new lesions, allowing more rapid monitoring and earlier intervention. Future studies should determine which lipids correlate with MRI measures of demyelination, and investigate whether CE(16:0) and MIF track with progression and therapeutic response in longitudinal MS cohort studies.

## supplementary material

10.1136/jnnp-2024-333774 online supplemental figure 1

10.1136/jnnp-2024-333774 online supplemental table 1

## Data Availability

All data relevant to the study are included in the article or uploaded as supplementary information.
