# Supplementary figures and images for: CSF sphingolipids are correlated with neuroinflammatory cytokines and differentiate neuromyelitis optica spectrum disorder from multiple sclerosis
Source: J Neurol Neurosurg Psychiatry. 2024 Jun 6;96(1):e333774. doi: 10.1136/jnnp-2024-333774 (PMC11672031; doi:10.1136/jnnp-2024-333774)

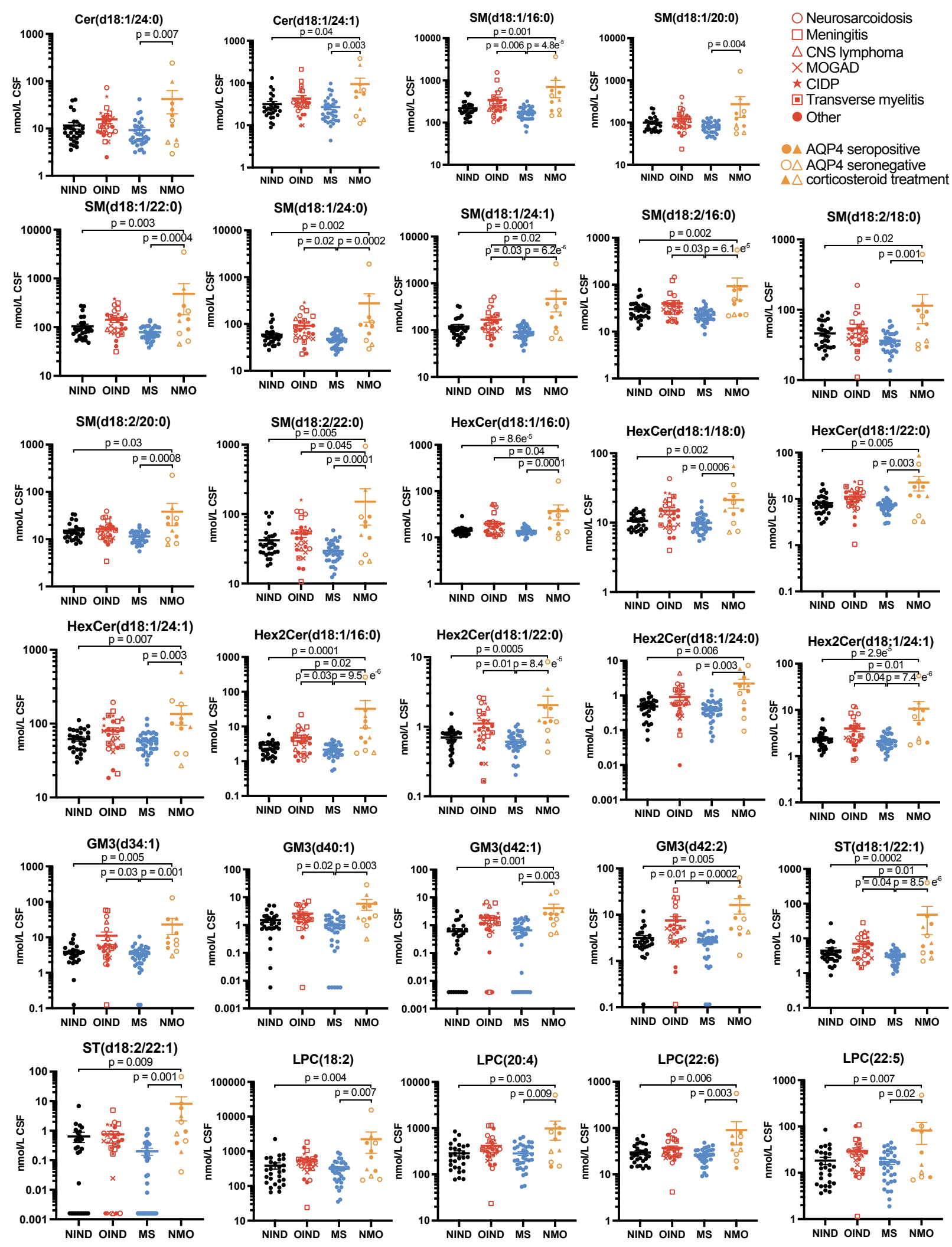

Supplement: online supplemental figure 1 [file jnnp-96-1-s001.pdf]
